# Parent perspectives of children with selective mutism and co-occurring autism

**DOI:** 10.1080/20473869.2023.2173835

**Published:** 2023-02-03

**Authors:** S. Keville, P. Zormati, A. Shahid, C. Osborne, A. K. Ludlow

**Affiliations:** Department of Psychology, Sport and Geography, University of Hertfordshire, College Lane, Hatfield, Herts, United Kingdom

**Keywords:** Selective mutism, autism, parent, child, advocacy, diagnosis

## Abstract

Selective mutism (SM) and autism frequently co-occur together, exacerbating social communication deficits and associated anxiety. However, professionals have lacked a readiness to diagnose SM and autism together, making the need to understand parental experiences of caring for a child with SM and autism crucial. The current study utilised Interpretative Phenomenological Analysis (IPA) to explore parents’ experience of caring for children with SM and autism. Semi-structured interviews were conducted with eleven mothers and one father of children aged between 5 and 18 years. All children were diagnosed with SM and had undertaken, or were currently undertaking, an autism diagnostic process. Analysis of the transcripts resulted in the following themes: Complexities from co-occurring issues; The overwhelming impact of SM; The diagnostic journey; Finding solutions and advocacy. Judgements and minimisation of symptoms from educational and healthcare systems exacerbated delays in diagnosis preventing appropriate intervention. The complexities of caring for a child with SM and autism, alongside wider misunderstandings, exacerbated parental stress, impacting the family. Parental advocacy and safe environments provided opportunities for children to better manage contextually based mutism. Improvements in identification and compassionate understanding from wider systems by involving parents as key stakeholders are essential to improve this situation.

Selective mutism (SM) is a condition with a typical onset in childhood. It is characterised by an inability to speak or respond in certain social environments and certain situations (for example, at school or with unfamiliar people), whilst having the ability to speak in other places and with other people (for example, at home and with parents) (American Psychiatric Association (APA), 2013). Moreover, to gain a diagnosis of SM, the situational lack of speech needs to be present for at least one month, interfere with general functioning, in addition to being differentiated from communication difficulties and a lack of language competency (APA, 2013, World Health Organization 2018). Research is beginning to recognise that symptoms typically associated with autism, may also be present in some children with SM (Muris and Ollendick 2021).

Diagnostically SM is classified as an anxiety disorder due to its co-existence with anxiety disorders in general (Cunningham *et al*. 2006, 2004), but more specifically with social anxiety (Melfsen *et al*. 2006, Vecchio and Kearney 2005), which remains the prominent co-occurring disorder in SM (Schwenck *et al*. 2021, Driessen *et al*. 2020). Indeed, children with SM have been shown to display comparable levels of anxiety to children with social phobia and other anxiety disorders (Levin‐Decanini *et al*. 2013, Vecchio and Kearney 2005). Nevertheless, SM can be differentiated from social anxiety by its earlier onset, and the increased likelihood of symptoms of SM remitting (Sutton 2013).

While SM is currently listed as an anxiety disorder, it has been suggested that the mutism associated with SM may conceal an underlying developmental condition due to its multifaceted and heterogeneous nature (Kristensen 2000). For example, several studies have highlighted high levels of co-occurring autism in children diagnosed with SM, with 62.9% of children diagnosed with SM being eligible for an autism diagnosis (Steffenburg *et al*. 2018). Autism is a neurodevelopmental disability marked by deficits in social communication, and restricted and repetitive behaviours or interests (APA, 2013), which may increase the complexities experienced within social situations for autistic individuals (Bellini 2006) and generate anxiety when interactions are required (Spain *et al*. 2020). Indeed, compared to controls, there is a higher rate of anxiety disorders within autism (van Steensel and Heeman 2017), particularly social anxiety (Spain *et al*. 2018). Importantly, rather than individuals being disinterested in social situations, studies on social anxiety and autism suggests anxiety drives avoidance (Maddox and White 2015), or engagement in camouflaging or stimming (a repetitive movement or vocalisation, often used for self-soothing) (Spain *et al*. 2020).

Due to the previous autism-related exclusion criteria required for an SM diagnosis, the prevalence of autism with SM has likely been vastly underestimated, leading many researchers and clinicians to ignore the co-occurrence of both conditions (Simms 2017). Therefore, much of the research addressing associations between SM and autism have been difficult to interpret especially as more extreme cases of autism are often excluded. For example, Andersson and Thomsen (1998), and Kristensen (2000) found 8.1% and 7.4% of the children in their studies fulfilled the criteria for Asperger’s syndrome respectively (an earlier name now incorporated under the autism spectrum term). Although Kristensen and colleagues found that the prevalence of autism rose to 25.5% when teachers’ ratings were considered, Klein *et al*. (2019) study using the more recent diagnostic criteria, found 80% of a sample of 42 children (aged 2–13 years) scored above the cut-off on the autism probability index of the Behavior Assessment System for Children (BASC; Reynolds *et al*. 2015).

Autism behaviours may also overshadow SM behaviours in some children, with social difficulties, including social communication and social motivation, identified in both groups of children (Cholemkery *et al*. 2014). Therefore, research to date addressing SM and autism may only reflect the individuals with autism who present with pervasive SM behaviours in specific contexts, such as school, and which are noticeable enough to be assessed (Schwartz and Shipon-Blum 2005, Ludlow *et al*. 2022). Consequently, SM may not be identified in some children with autism due to its lack of presentation in other contexts, or where autistic behaviours are more prominent (Steffenburg *et al*. 2018). Yet when they do co-occur, they are more difficult to treat (Valaparla *et al*. 2018).

With autism being a lifelong disability, emerging complexities, such as SM, can make the process of identification, diagnosis and support stressful for parents; indeed, difficulty accessing services can be challenging for parents meeting the needs of autistic children with co-occurring issues (Vohra *et al*. 2014). Further, given communication deficits in autism, parents often communicate on behalf of their child, yet there are barriers within this advocacy role including perceived stigma around an autism diagnosis, additional time commitments required parenting a child with autism, financial challenges, as well as a lack of service availability, knowledge and support from service providers (Smith-Young *et al*. 2022). Obtaining a diagnosis for SM has also been shown to be difficult for parents. For example, while onset of SM is usually between the ages of 2 and 5 years (Cunningham *et al*. 2006, Kristensen 2000), SM often remains undiagnosed until later, or not at all (Muris and Ollendick 2021).

Currently, there is no guidance on the process for diagnosis and intervention for SM and autism (McKenna *et al*. 2017), and minimal research on the co-occurrence of SM and autism resulting in SM symptoms being labelled as autism-related difficulties around social communication (Simms 2017). Likewise, as the inability to effectively communicate takes attention away from other autistic features, autism can be missed in SM (Ipci *et al*. 2017). This is concerning as SM is not rare, with estimated prevalence rates between 0.71% (around one in 143 children; Bergman *et al.* 2002) and 1.9% (approximately one in 50 school-age children; Kumpulainen *et al*. 1998). Therefore, given the complexity of symptoms and current lack of clinical guidance on diagnosis and treatment for children presenting with both conditions, this study aimed to understand the impact of SM and autism from a parental perspective.

## Materials and methods

### Research design

Due to the need for an exploratory ideographic focus within an under-researched area, this study utilised Interpretative Phenomenological Analysis (IPA) to provide a detailed and in-depth analysis and exploration of the lived experiences of parents caring for a child with SM and co-occurring autism (Smith *et al*. 2009). IPA standards for ensuring quality within qualitative research were followed throughout (Nizza *et al*. 2021).

### Participants

A purposive sampling method was used to recruit parents of children with a diagnosis of SM and possible autism through the SM Information and Research Association (SMIRA); 23 parents expressed interest and gave their contact details. In total 12 parents from the United Kingdom responded when contacted and participated in semi-structured interviews. Based on caregiver report, all the children met the criteria of having a formal clinical diagnosis of SM; and all met the inclusion criteria of having a child with autism or were currently undertaking an autism diagnostic process.

The sample consisted of 11 mothers and 1 father (Table 1); parents were aged between 43 and 56 years (*n* = 9 responses; mean = 50; S.*D* = 4.77). The participants’ children were aged between 5 and 18 years (mean = 12.66; SD= 4.6). The mean age for SM diagnosis was 6.33 years (S.*D* = 5.25) and the mean age for assessment or diagnosis with autism was 10.27 years (S.*D* = 4.5).

**Table 1.** Participant and child demographic information.

Participant	Age	Gender	Occupation	Marital status	Ethnicity	Age of Child (yrs)	Child’s gender	School setting	Age assessed or ¹ diagnosed with autism	Age ¹diagnosed with SM	Child’s clinical ¹diagnosis of other conditions
Eva	47	Female	Stay-at-home parent	Single	White	14	Male	Secondary	In process	3	^4^SAD
Nichole	53	Female	Carer	Married	White	16	Male	Secondary	8	3	^5^GAD
Stacy	50	Female	Self-employed	Married	White	15	Transgender (Male)	Secondary	11	12	Anxiety
Charlotte	56	Female	Stay-at-home parent	Married	White	16	Female	College	In process	4	²ADHD, anxiety, panic, depression
Chloe	-³	Female	-³	-³	White	17	Female	Secondary	14	15	^4^SAD; gastrointestinal problems
Linda	53	Female	Stay-at-home parent	Civil partnership	White	18	Male	College	15	17	None
Jack	45	Male	Carer	Single	White	8	Male	Primary	6	6	None
Olivia	43	Female	Tutor	Married	White	7	Female	Primary	4	4	Tourette’s Syndrome
Layla	47	Female	Stay-at-home parent	Married	White	13	Male	Secondary	11	2	Language delay, learning disability, sensory impairment
Claire	-³	Female	-³	-³	White	5	Male	Primary	In process	2	Hypermobility
Avery	56	Female	Stay-at-home parent	Married	White	16	Female	College	In process	4	²ADHD, ^4^SAD, ^5^GAD, phobia, headache
Jennifer	-³	Female	-³	-³	White	7	Female	Secondary	7	4	Language delay

^1^ Assessed/diagnosed by healthcare professional.

^2^ Attention deficit hyperactivity disorder.

^3^ -no response.

^4^ Social anxiety disorder.

^5^ Generalised anxiety disorder.

### Instrument and data collection

Demographic data were collected (Table 1), and experiences explored through semi-structured interviews. Utilising quality criteria (Treharne and Riggs 2015, O'Brien *et al*. 2014), the interview schedule was developed and refined by the research team by consulting SMIRA and others with lived experiences of caring for a child with SM/autism. Questions included:

1. In what ways does selective mutism and autism impact on your child?
2. What are your experiences of the communication between school, health care providers and your family regarding your child’s SM, autism and wellbeing?
3. Can you describe the moment when you first became aware of your child’s difficulties speaking to others in some situations and not others?
4. How did you find the assessment process in relation to SM?
5. What is the best advice you would give to parents about managing your child’s SM alongside school and social activities that involve communicating with others?

Interviews were audio/video recorded via an online video conferencing platform and transcribed by the second and third authors. Interviews lasted 34 – 119 min (mean = 57.83; SD = 23.77).

### Ethics

University of Hertfordshire Health, Science, Engineering and Technology Ethics Committee with Delegated Authority granted approval (aLMS/SF/UH/04545(1)). Participants were provided with study details, including publication using anonymised data and their right to withdraw; and written informed consent obtained. Researchers’ maintained confidentiality throughout, pseudonyms were applied, and recordings deleted after transcription. Following interviews participants received a debrief sheet including links for additional support.

### Data analysis

Each transcript was read multiple times by the second and third authors; reflexive conversations were conducted throughout with the first and last authors in accordance with quality guidelines for IPA (Nizza *et al*. 2021) and qualitative research (O'Brien *et al*. 2014, Treharne and Riggs 2015). Reflexivity also involved the use of reflective notes to explore and acknowledge researcher bias. Emergent themes and interpretations were noted alongside divergent and convergent themes to highlight the unique experience of each participant (Nizza *et al*. 2021). To ensure credibility, triangulation occurred through consultations with clinicians and those with lived experiences, with the study adopting the principles of co-production (Voorberg *et al*. 2015). Consequently, the concept and development of the project, research team participation and authorship of the paper included those with parental and/or individual lived experiences of SM and autism; alongside research students and a researcher experienced in neurodevelopmental conditions. All reviewed and agreed themes and quotes used within the paper. Participants were sent the theme table of superordinate themes and subordinate themes; no amendments were requested.

## Results

Following the IPA analysis, four superordinate themes emerged with several subordinate themes (see Table 2).

**Table 2.** Superordinate and subordinate themes.

Superordinate themes	Subordinate themes
Complexities from co-occurring issues	Interactions between sensory overload, SM and autism
	Fatigue, communication freezes and shutdown
The overwhelming impact of SM	Affects every aspect of child’s everyday life
	The impact on siblings and the family
	The emotional burden on parents
The diagnostic journey	Navigating the minefield
	A lengthy process: hindered development and hidden abilities
Finding solutions and advocacy	Taking control and finding solutions
	Assistance and advocacy

### Complexities from co-occurring issues

This theme illustrated how complexities from overlapping symptoms derived from people, the environment, and sensory overload impacted the children and their ability to communicate.

#### Interactions between sensory overload, SM and autism

All parents commented on the negative impact people, the environment and sensory overload had on their child, Stacy stated:

The environment is the reason why mainstream school …may not…have suited him, because noise has a big impact on him…smells, busyness of an environment so lots of people, lots of people usually means lots of people talking that means noise.

The triple repetition of ‘lots of people’, emphasised the overwhelming environmental challenges generated by people within mainstream school. Similarly:

The place was busy, and those people talk, and it was a bit smelly. But she couldn’t communicate that with me, and she was getting angry with me that I was trying to speak to her to ask her what I wanted her to do. (Olivia)

The diverse sensory onslaught seemed to trigger anger in Olivia’s daughter, perhaps due to distress, shutting down her ability to communicate. Everyday tasks were a struggle, as noted by Chloe, whose daughter was ‘struggling to start a conversation, I think it’s tied in with both autism and SM’; and Charlotte stated:

I think she recognises obviously…that she has selective mutism. And that stops her interacting because she gets too scared and almost like she, her voice like freezes, so she can’t, like reply. And then I think also, she’s, like, really scared that she’ll say the wrong thing. So that stops her as well.

Her daughter’s ‘voice…freezes’ seemed deeply interlinked with fear when she repeated ‘scared’; for some parents being unable to address their child’s sensory issues made them feel ‘helpless’, as Jack noted: ‘It’s like a bit helpless that I want to help them but there’s nothing I can do about the noises’.

In stark contrast away from the confines of school and demands therein, some children seemed almost liberated; Eva smiled when she stated: ‘When we go on holiday it’s as if his SM is lifted, as if he never had it’.

#### Fatigue, communication freezes and shutdown

Participants described how fatigue impacted their child’s ability to communicate: ‘I might get a grunt because she’s so tired’ (Jennifer); and: ‘It’s really bad if he’s tired…you very rarely get anything out of him’ (Linda). Olivia extended this to include stress: ‘Once she’s stressed or really tired and if you’re out and about and you’re in a shop she won’t be communicating but she also won’t want anyone else in the family to communicate either’. Needing all interactions around her to cease, seemed a protection from the potential demands placed upon her.

For others there were complex interactions between people, sensory stimuli, autistic symptoms and communication shutdown; Jennifer recounted an interaction when her daughter asked why her Grandma could not do something:

“…because I’ve got a pain and it really hurts my arm” and [daughter] said, “Where?”, and instead of [Grandma] touching her own arm, she touched [daughter]’s arm, and [daughter] shutdown instantly stopped speaking just because she’d been touched, because she wasn’t expecting….

This ‘shutdown’ when unexpectedly touched was instantaneous, resembling an automatic response, beyond thinking. In the context of explaining her son’s difficulties at school, Layla stated he ‘is fine at home’ but would shutdown when returning to school after breaks: ‘…he’ll stop communicating with us at all, and he goes really upset and depressed’. This seemed difficult for her to witness when she concluded: ‘…it’s horrible’.

Many participants noted early markers of this shutting down response before their child had developed language. For example, Claire stated her son would: ‘…be very happy and sociable if he would go up to them, but if they were to force that upon him, he would shutdown even before he could speak’. Claire further noted that her son ‘is only mute in formal situations where there’s some kind of an expectation upon him’.

Clearly parents highlighted individualized stressors triggering a shutdown response, which was also generated by COVID-19: ‘…this COVID, and the lockdown and everything that caused him a lot of distress. And that’s really made him shutdown’ (Linda). Nevertheless, some benefits emerged for some children from COVID-19 related adaptations simplifying social interactions at school:

…the playgrounds been in bubbles…she’s just loved it and she just said, “I just don’t want to go back to school after because the playground is going to be too busy and I’m not gonna be able to speak to anybody”. (Olivia)

### The overwhelming impact of SM

This theme captured the pervasive, and more direct impact of SM on parents’ children, their families and themselves.

#### Affects every aspect of child’s everyday life

Most parents reported their child’s SM affected every aspect of life:

Epically, so it affects her everyday life and her ability to function…with everyday tasks…to be able to do normal activities, what we would consider normal activities and her ability to function in school and achieve academically…her own understanding of yourself. Yeah, yeah, every aspect. (Olivia)

The term ‘epically’ denoted an immense, pervasive impact of SM across all domains; the impact on everyday basic tasks contrasted with ‘normal activities’ expected in neurotypical development.

Indeed, some parents commented on the more complex relational impact of SM: ‘The ability to be able to make friends because sometimes I think she feels like lonely, and she would like to have friends…but it causes her stress…like even to text them’ (Charlotte). One could sense how SM trapped Charlotte’s daughter in a ‘lonely’ position due to the demands relationships placed on her. Charlotte went on to highlight the ‘stressful’ fear-inducing nature of social interactions: ‘…she finds it really stressful, and she doesn’t know what to say…she gets really overwhelmed and scared when she does have interactions’.

Further, dealing with peer behaviours at school was particularly aversive for some, made worse through vocal freezing seen in SM: ‘…a girl locked her in the toilet, and she couldn’t scream and ask for help. She had lots of negative experiences at school’ (Avery). Additionally, Jack directly implicated how his son’s communication issues meant others’ bullying behaviour remained unaddressed: ‘…some lads kicking him under the table for weeks…and because he can’t talk, they got away with it’. One can only imagine the longer-term disempowerment derived from the inability to voice distress; also, for the parents who seemed powerless to immediately address the issue.

#### The impact on siblings and the family

Most parents reported impacts on the family and siblings derived from the complexities of their child’s SM alongside other conditions:

I don’t think moving forward with…difficulties with being autistic and having selective mutism and she also has Tourette’s as well…life will never be…something where we can go to every social function and be able to do the things that people anticipate you can do, so I do think we do keep ourselves shut away a little bit still. (Olivia)

Compared to other people’s abilities and expectations, Olivia is clear on the social limitations her daughter’s neurodiversity presented, keeping the family ‘shut away’. Olivia also acknowledged the social impact on her other child:

When we are out socially, we can’t interact with anybody and it’s definitely had a real negative impact on my youngest…she really struggles….at school, socially, because she…just hasn’t had the same interaction…people aren’t able to come to our house… yeah, it’s tricky, it’s tricky.

Nichole highlighted the inability to sustain a career: ‘I was a midwife I had to give up my job…I’ve never been able to go back because my days are taken up with what’s our next battle annual review each year’. There was an implication that accessing care for her son’s wellbeing was a full-time job, involving a perpetual battle at the ‘annual review’, an Education, Health and Care Plan (EHCP) procedure used to support school age children with additional needs.

#### The emotional burden on parents

Accessing provision seemed an ongoing struggle for parents, often necessitating stepping away from standard provision: ‘…doctors weren’t particularly interested, so we just decided to go straight down the private route, quicker and easier’ (Jennifer); and ‘…in the end we paid for a private assessment, because it was 18 months and still hadn’t heard from CAMHS [Child and Adolescent Mental Health Service]… the health care system has been a joke’ (Claire). Ultimately, most parents were emotionally impacted by managing wider issues alongside their child’s difficulties, one notable issue involved difficult interactions with the local authority (LA):

I almost feel like I don’t think I don’t know whether I’m angry I don’t know whether I’m just in despair over them and thinking okay you’re all useless. It’s took us, it took me a while to come to that conclusion. (Nichole)

The language evoked a sense of incredulity that those tasked for assessing and providing provision were ‘useless’, conjuring intense emotions. Coming to this realisation seemed difficult in the context of managing her child’s complex needs and wellbeing.

### The diagnostic journey

This theme captured a difficult journey as parents navigated the process of making sense of their child’s needs, alongside accessing referrals, diagnoses and support.

#### Navigating the minefield

Some parents experienced a lack of understanding from relatives, teachers and even health-care professionals when they tried to explain their concerns:

I think there it’s just lack of understanding, and I think when someone who is meant to be…so close to you…and a lack of understanding or willingness to understand, at a time when I think…you probably just need people to be supportive…you’d generally think well if they feel…and think like that, what does the rest of the public…think really? (Olivia)

Olivia implicated the importance of close relationships to provide a more compassionate response during times of need. Indeed, when explaining SM to a health-care professional Eva laughed, perhaps at the irony of a child expert minimising it: ‘… “well, selective mutism is nothing, you know”, and I was like, you are a consultant at CAMHS’ (laughs). Others found ‘doctors weren’t particularly interested’ (Jennifer). Sometimes misunderstanding was pervasive and damaging, to the point where a teacher believed they could ‘cure’ SM by heightening focus on it:

….super focus on her difficulties of not speaking and her teacher wanted to cure her you know she nearly had a breakdown she seriously was so getting ill; we have a picture of her, she looks a shadow of a poor child. (Avery)

Others experienced mismanagement from family members: ‘…his mom’s sister got him in the hallway, wouldn’t let him pass and go, “you’re not getting past till you talk to me”. No, that’s not the way to deal with it’ (Jack).

This lack of understanding seemed interlinked with mistaken beliefs that SM was due to indulgent parenting: ‘Other people just probably think you’re not a very good parent, I don’t know, you’ve let your child get away with a lot, they’re just spoiled’ (Charlotte); and: ‘…a lot of it was a reflection on our parenting when she was very young, people assumed that how our parenting was creating a child that wasn’t speaking’ (Olivia).

Others had mixed responses to SM dependent on willingness to understand and adapt; Claire stated some viewed her son as ‘controlling, manipulative and not wanting to talk’, whilst other families were ‘pretty good and they do try to avoid putting him on the spot’.

Olivia repeated several times that misunderstandings and minimisation could derive from the term ‘selective’:

…it has an unfortunate title, I think. The word selective makes people assume that she’s doing it, she’s selecting to do it or not, and it’s been tricky…it’s caused a lot of conflict within…family members having a lack of understanding.

EHCP procedures and wider systems similarly impacted participants negatively:

…they did a really terrible assessment, basically, which I expected so that’s when I got another EP [educational psychologist]…again to do a thorough assessment…[the LA wrote] “Mrs Nichole is amassing another round of private reports”, and it was basically saying if school don’t support us [LA] we’re going to lose this tribunal….the head was bullied. (Nichole)

It seemed Nichole was in a battleground along with the school, with the LA undermining parental attempts to access appropriate assessments. Trying to access support required resilience and strength to withstand the onslaught: ‘You’ve got to have this kind of rhino skin to fight; everything’s a fight to get a GP to refer you, to get the school to refer you, to get the SALT [speech and language therapist] to refer you’ (Eva).

#### A lengthy process: Hindered development and hidden abilities

Alongside systemic roadblocks, misunderstandings surrounding SM meant it took time to comprehend; subsequently, for some parents getting a diagnosis and accessing support was a lengthy process:

…over the years, it’s just developed into normal SM, all his traits are very ASD [autism spectrum disorder], but no one can assess that…which is why it’s taken over 10 years for us to get an assessment…He got diagnosed when he was three…until he was 13…we couldn’t find a SALT that had specialism in selective mutism. (Eva)

Most parents seeing their children in a wide variety of situations could see beyond their child’s issues towards hidden skills and abilities which SM suppressed, or even took away: ‘It’s not really fair on him…he’s a lovely child. He’s that like, kind and helpful’ (Jack); and: ‘He was actually really talented in terms of performance and acting. And then from about nine that kind of all stopped…more introverted…lots of worry, lots of anxiety, wanting to kill themselves, attempted to kill themselves’ (Stacy). Clearly, the longer-term impact on wellbeing could increase risks of suicide.

With variability of SM in different situations and with different people, these abilities potentially complicated any assessment process. Likewise, the inability to communicate, meant some children could not be diagnostically assessed:

The diagnosis process was a lot longer for us, because…they had to witness [child’s name] speaking…with them to be able to diagnose autism, because without any communication…they wasn’t able to diagnose autism….it was a long, long process, for us. (Olivia)

The repetition of ‘long’ emphasised this duration; one can only imagine the potential impact this had on accessing support earlier; which was already hindered by issues within the system.

### Finding solutions and advocacy

This theme highlighted the ways parents adjusted to help their child; solutions needed careful consideration alongside direct action.

#### Taking control and finding solutions

Most parents took control to access the right support for their child: ‘God we argued…I was saying he needs them. You can’t treat selective mutism at that degree, at his age, unless it’s therapy and meds together’ (Eva). This seemed a common theme with some parents organising their own assessments to access LA provision: ‘I realised that in order to get an EHCP and get the support that my son needed, I needed to arrange my own assessments because local authority, were not going to do that’ (Stacy). There was a sense of cat and mouse - without assessments to demonstrate needs, provision was not allocated; yet LA’s ‘were not going to do’ assessments, leaving parents in a quandary. Whatever route was taken required ‘hard work…and persistence…also being able to afford to pay for an assessment process….many people are not able to find the right resources’ (Stacy). It seemed that only parents with knowledge and the financial means to pay for necessary assessments could take this route, even then this was not always successful.

Adjustments for communication deficits were continual, and parents offered essential advice. Eva emphatically stated: ‘So I figured out very early on, you never shout at him…if you’re cross with him…any time’; and Jack suggested managing communication freezes through: ‘…the more of a relaxed atmosphere is the better…You know, don’t try to push them into things that [they] don’t want to do’.

#### Assistance and advocacy

Becoming an advocate for their child seemed essential:

I help and become his advocate. And I will interpret for him, and I will encourage him to be able to use their voice. And if he can’t use their voice, then he will be encouraged to write what they need to say. (Stacy)

To teach independence, Stacy negotiated a tricky balance of advocating whilst supporting her child to communicate vocally or through writing; yet invariably this was impacted by others’ judgements:

We went to a party…she was completely unable to communicate…it’s really stressful because again there’s that you don’t want to feel…people are…looking and judging…I try not to you know kind of speak for her too much but certainly when she was much younger I did step in a lot. (Olivia)

This negotiation was ‘stressful’, yet regardless of judgements, Olivia’s daughter needed her parent to fulfil this role. Further, Claire highlighted the dilemma between helping or hindering learning:

…always very hard knowing how much to step in and what point do you rescue, and actually, are you being any help by rescuing because you rescue them forever, then they never learned to do it.

Perhaps initially, their children needed a familiar person advocating and ‘rescuing’ them until they felt safe enough to do this themselves. Indeed, this familiarity was crucial in school settings: ‘if there’s new teachers there then she definitely wouldn’t speak to anybody that she doesn’t know’ (Jennifer). Indeed, Avery indicated the differing qualities for a child with SM needing parental support: ‘…it’s not as in separation anxiety as like normally…when they’re overly emotionally attached’. Avery went on to highlight how anchorless a child with SM and autism could be without a familiar, safe and trustful person advocating and supporting them: ‘…it’s because the caregiver isn’t there anymore’. Indeed, Avery articulated that this was akin to providing aids for those with physical disabilities:

…If you had a hearing aid or…glasses and someone took it away, how anxious you would be because you don’t have them; and it’s like how they feel towards the main caregiver because that…source and protection is gone.

## Discussion

Despite the suggestion of a high co-occurrence of SM and autism (Steffenburg *et al*. 2018), this is the first qualitative study exploring parental experiences of caring for a child with co-occurring SM and autism. The pervasive impact of SM and autism affected all aspects of life, including their child’s ability to form friendships, find their voice, and take part in activities; it also impacted siblings’ activities and social development. Some parents adapted their lives or gave up careers to focus on the management and advocacy required for their child to gain independence. Parents additionally highlighted a complex interaction between SM and autistic symptoms, including anxiety and sensory challenges, which exacerbated their child’s communication difficulties (Muris and Ollendick 2021).

The overwhelmingness of environmental sensory overload at school meant many of the children experienced communication freezes and shutdown. The term shutdown describes a response to stressful circumstances when individuals with autism cannot control sensory sensitivities and social demands, for example, at school (National Autistic Society (NAS), 2022), and is a complex response to autism related stressors (Shah 2019). In the current study, three parents similarly used the term shutdown; and akin to the findings from other studies, some parents described shutting down processes involving communication freezes when overwhelmed, fatigued or during sensory overload (Keville *et al*. 2021).

It is well documented that individuals with autism have sensory sensitivities due to abnormal auditory, visual, touch and oral sensory processing (Kern *et al*. 2006), with abnormal cortical auditory processing implicated in exaggerated behavioural responses to sounds (Boddaert *et al*. 2004). These sensory hypersensitivities are associated with anxiety (Uljarevic *et al*. 2016) and have a significant role in the social and communication problems seen in autism, impacting engagement in social interactions (Thye *et al*. 2018). Further, children with SM have abnormalities in auditory regulation and monitoring of self-vocalisation (Muchnik *et al*. 2013), potentially making their voice sound strange and resulting in the avoidance of talking (Bar-Haim *et al*. 2004, Vogel *et al*. 2019). All parents stated their child experienced sensory sensitivities and overload in a wide range of sensory domains, and it has been noted that sensory avoidance within SM may be indicative of undiagnosed autism in children carrying an SM diagnosis (Ludlow *et al*. 2022).

Consequently, given that intolerance of uncertainty is considered a predictor of children’s sensory sensitivities (Neil *et al*. 2016), within new environments and with new caregivers, parental accounts highlighted that it was important to initially provide children with a consistent, trustful parental presence. This ensured children felt safe to develop later independence in communication. Yet, unlike parental advocacy with autistic children (Smith-Young *et al*. 2022), parents providing a voice for a child with SM and autism seemed to enhance a lack of understanding, possibly due to the understandable caution clinicians have around negative impacts from family accommodation, that is, over or under managing their child’s daily routines as a means of minimising increases and/or maintenance of their child’s anxiety (Strohmeier *et al.* 2020). Interventions for anxiety often focus on minimising avoidance through exposure to feared situations/objects; in this context this might involve misguidedly recommending the removal of the parental voice, to enable children to find their own voice. Yet, for parents of children with SM and autism whose children shutdown, communicating ideas of accommodation can add to the parental burden derived from negative judgements about their parenting. Instead, parents noted parental advocacy was crucial in accessing support and protection for their child, alongside facilitating their child to gain vocal independence. The added complexity and pervasive misunderstandings of SM within autism may further contextualise the added burden of caring for a child with SM and autism, given the associated communication deficits in autism.

With SM impacting all areas of life, this lack of understanding extended to close relatives highlighting a frustrating and stressful experience when attempting to access compassionate understanding. Private referral routes for diagnosis and support seemed to fare better for those who could afford them, though may disadvantage those who cannot. Regardless, accessing referrals, diagnosis and support was a lengthy process for some parents, as has been reported by parents with autism and other co-occurring disorders (Brookman-Frazee *et al*. 2012, Adamson *et al*. 2020), with complexities exacerbating misunderstanding by clinicians. Perhaps judgements may be exacerbated by the term *selective*, which denotes choice around when to communicate, and with whom. Indeed, children who experience SM are frequently misperceived as being quiet, shy, and in some cases oppositional (Johnson *et al.* 2015). However, parents described SM as a communication freeze triggered by the environment and people – it was a contextually (and sometimes physiologically) based reactive response rather than an actively chosen one. Moreover, it is now recognised that SM and associated SM symptomology can be identified in families of children with this condition (Remschmidt *et al*. 2001), with parents showing higher levels of shyness, social anxiety and engagement in solitary activities (Kristensen and Torgersen 2001, 2002), potentially highlighting underlying familial factors contributing to withdrawal in social contexts.

Rather than aiming for generalised claims, IPA aims for case-to-case transfer generalisation (Treharne and Riggs 2015). Nevertheless, despite all parents being white and 11 were mothers, this study had a large sample for an IPA analysis retaining the ability to explore the data in depth (Smith *et al*. 2009). Further, diagnoses were self-reported and whilst all participants had a child with SM, due to the later age of diagnosis for autism, some children were currently in the process of undertaking an autism diagnosis. Nevertheless, parents described a similar experience to parents whose children were diagnosed. Future research could utilise the Autism Spectrum Screening Questionnaire and the Selective Mutism Questionnaire to corroborate self-reported diagnoses (for example, Ludlow *et al*. 2022); and explore parental experiences of SM and autism within other demographic populations. Addressing these limitations seems critical when exploring access to identification and diagnostic opportunities for SM and co-occurring autism.

### Conclusion and clinical implications

For parents, the experience of caring for a child with SM and autism required strength and resilience due to the complex interaction between their child’s diagnoses and widespread misunderstanding impacting parental wellbeing. Communication freezes meant parents had to advocate for their child which exacerbated judgements and minimisation, resulting in ongoing battles to access assessments and support. Furthermore, the diagnostic process may have been made more complicated by, not only the reliance on self-reports and/or parent/teacher interviews often used to determine diagnosis, but also the children’s varying speech and language difficulties and associated co-occurring diagnoses. Nevertheless, parents of both children with SM and children with autism, often struggle to access support for their children in a timely manner, if at all (Johnson *et al*. 2015, Vohra *et al*. 2014).

While no studies have addressed parents’ perspectives of these dual diagnoses, parental perspectives are crucial for a timelier diagnosis. Personal experiences can aid understanding of the early signs and development of SM and co-occurring autism, and it may help in providing clinicians with a more tailored, person-centred approach. Moreover, previous studies focussing on understanding the needs of parents of autism during the assessment process, highlights the importance of communicating with a professional who listens, recognises strengths and difficulties, and who can provide a sense of hope (Abbott *et al.*, 2013, Woodgate *et al*. 2008). Indeed, families place more importance on having an opportunity to speak about the diagnosis rather than the provision of information per se (Rabba *et al*. 2019).

Given the earlier onset of SM in this sample compared to autism, SM seemed to be an earlier marker of a neurodevelopmental or underlying condition necessitating the exploration of underlying conditions underpinning greater sensory sensitivities in these children and more severe SM symptomology (Ludlow *et al*. 2022); consequently, it is important to reframe this behaviour and remove underlying assumptions that it is deliberate or manipulative. Accordingly, given the lifelong role parents have in advocating for a child with autism (Smith-Young *et al.* 2022), valuing parental advocacy and involving parents as key stakeholders in policy development, assessment/intervention guidelines, training and education around contextually based mutism is essential for early recognition, diagnosis and the building of safe environments and child-led interventions. For example, involving the child too early in the diagnostic process with clinicians unknown to them, risks limiting access to available information. Moreover, putting pressure on the child to use their own voice too early could potentially lead to a stressful first encounter with those delivering treatment, increasing the likelihood of later disengagement (Furr *et al*. in press). Importantly, school-based intervention studies for children are more effective and less stressful for children with SM compared to those carried out in clinical settings (Mayworm *et al*. 2015), again implicating the importance of utilising known environments with familiar people for these children.

The literature highlights the desire of teachers to access training around the presentation and nature of SM from informed professionals (Lawrence 2017); this is vital given two-thirds of teachers in one survey did not associate SM with anxiety, let alone autism (Dillion 2016), despite both being highly co-occurring conditions. Therefore, the role of an educational/school-based psychologist may be essential in improving timely diagnosis and interventions for children with SM, both with and without autism (White and Bond 2022). Further, it is critical to create safe autism friendly environments (Roberts and Webster 2022) to enable children with SM and autism to find their own voice, as they progress into adulthood and beyond.
